# Uses of artificial intelligence in glioma: A systematic review

**DOI:** 10.3892/mi.2024.164

**Published:** 2024-05-20

**Authors:** Adham Al-Rahbi, Omar Al-Mahrouqi, Tariq Al-Saadi

**Affiliations:** 1 College of Medicine and Health Sciences, Sultan Qaboos University, Muscat 123, Sultanate of Oman; 2 Department of Neurosurgery, Khoula Hospital, Muscat 123, Sultanate of Oman; 3 Department of Neurology and Neurosurgery-Montreal Neurological Institute, Faculty of Medicine, McGill University, Montreal, QC H3A 2B4, Canada

**Keywords:** artificial intelligence, glioma, glioblastoma, machine learning, algorithm

## Abstract

Glioma is the most prevalent type of primary brain tumor in adults. The use of artificial intelligence (AI) in glioma is increasing and has exhibited promising results. The present study performed a systematic review of the applications of AI in glioma as regards diagnosis, grading, prediction of genotype, progression and treatment response using different databases. The aim of the present study was to demonstrate the trends (main directions) of the recent applications of AI within the field of glioma, and to highlight emerging challenges in integrating AI within clinical practice. A search in four databases (Scopus, PubMed, Wiley and Google Scholar) yielded a total of 42 articles specifically using AI in glioma and glioblastoma. The articles were retrieved and reviewed, and the data were summarized and analyzed. The majority of the articles were from the USA (n=18) followed by China (n=11). The number of articles increased by year reaching the maximum number in 2022. The majority of the articles studied glioma as opposed to glioblastoma. In terms of grading, the majority of the articles were about both low-grade glioma (LGG) and high-grade glioma (HGG) (n=23), followed by HGG/glioblastoma (n=13). Additionally, three articles were about LGG only; two articles did not specify the grade. It was found that one article had the highest sample size among the other studies, reaching 897 samples. Despite the limitations and challenges that face AI, the use of AI in glioma has increased in recent years with promising results, with a variety of applications ranging from diagnosis, grading, prognosis prediction, and reaching to treatment and post-operative care.

## Introduction

Glioma is the most prevalent type of primary brain tumor in adults, which is responsible for >80% of malignant intracranial tumors (1-3). Glioma tumors were categorized in the past into two categories based on their aggressiveness as follows: Low-grade glioma (LGG) and high-grade glioma (HGG) (4-6). Some LGGs are benign tumors, whereas HGGs are malignant tumors (7,8). However, the necessity for a new enhanced system arises as a result of rapidly increasing knowledge from clinical and molecular neuro-oncological research with high output. The fifth edition of the WHO Classification of Tumors of the Central Nervous System (CNS), published in 2021, focuses on the employment of intricate histological and molecular methods to determine the pathological diagnosis and grade of a tumor (9). Some authors prefer to use the term cancerous glioma as opposed to the prior term LGG, as major revisions to the current WHO classification have increased the importance of molecular diagnostics in the classification of CNS tumors (10). Currently, ‘glial, glioneuronal, and neuronal tumors’ are classified as a different family and are separated into six categories as follows: i) Adult-type diffuse gliomas; ii) pediatric-type diffuse LGGs; iii) pediatric-type diffuse HGGs; iv) circumscribed astrocytic gliomas; v) glioneuronal and neuronal tumors; and vi) ependymal tumors (11).

To prevent tumor recurrence rates and develop effective treatments, it is imperative to leverage artificial intelligence (AI) to comprehend tumor heterogeneity (12). This is based on ‘radiomics’, which typically consists of procedures and methods for extracting quantitative information from available imaging data (13).

The present systematic review discusses the uses of AI in glioma detection, grading, the prediction of the isocitrate dehydrogenase (IDH) genotype, O^6^-methylguanine-DNA-methyltransferase (MGMT) promoter methylation status, 1p19q codeletion, survival prediction, treatment response, pseudo-progression and progression, and the glioma functional network.

Glioblastoma (GB), previously known as GB multiforme (GBM) is one of the most aggressive types of brain cancer, which is characterized by its rapid development, poor response to therapy and low rate of survival (12).

AI is used in glioblastoma detection, for the prediction of the overall survival (OS) rate, and for determining the MGMT promoter methylation status, which is a mutation positively associated with an improved prognosis and temozolomide treatment response. AI is also used to determine tumor progression and pseudo-progression.

## Data and methods

**Figure 1:**
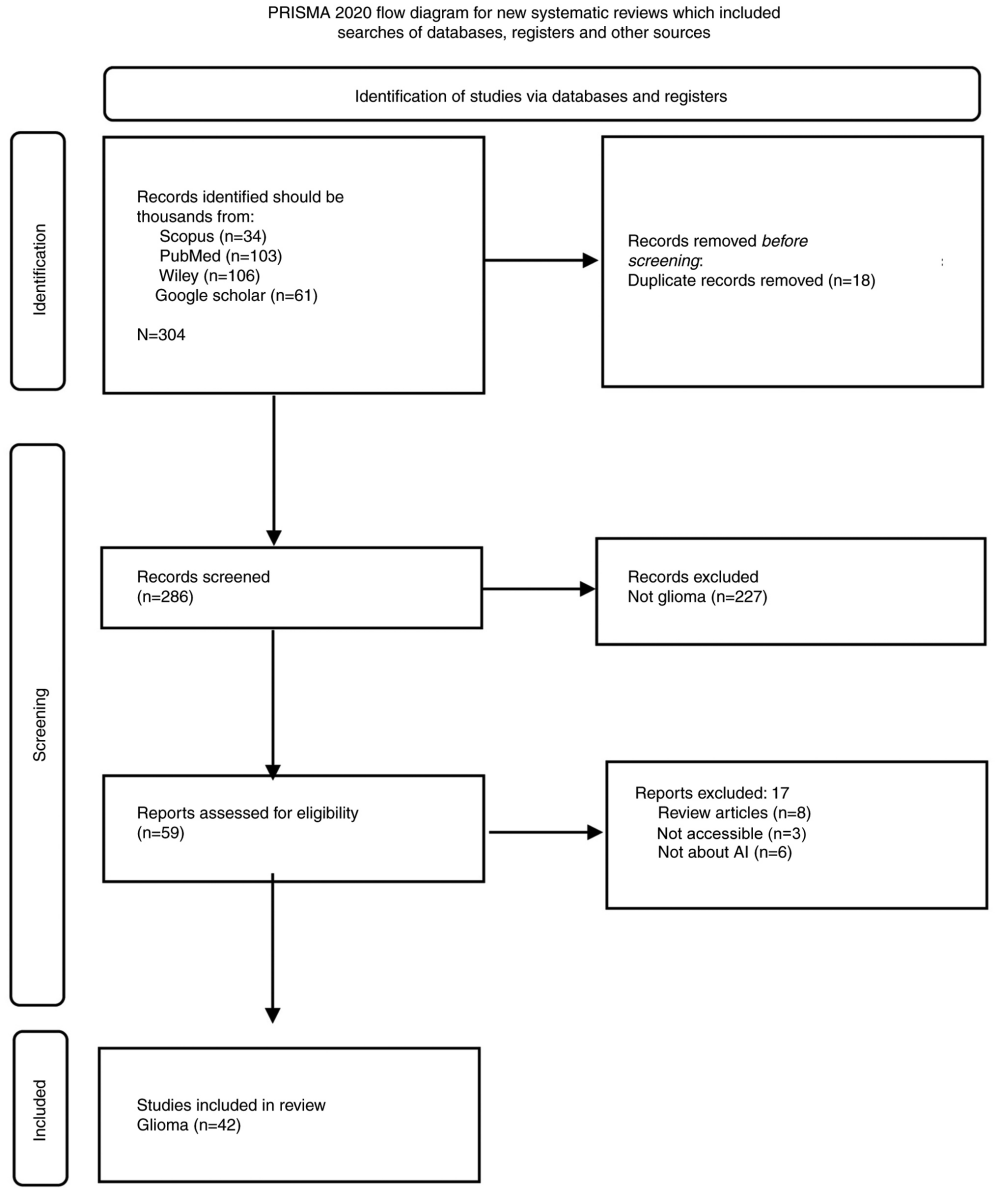
The PRISMA guidelines that were followed in the present study. PRISMA, Preferred Reporting Items for Systematic Reviews and Meta-Analyses. The guidelines used were according to the study by Page *et al* (67). For more information, please visit the following website: http://www.prisma-statement.org/.

The aim of the present study was to determine the trends of AI use in glioma and GB. For this purpose, a search is conducted in multiple databases including (Scopus, PubMed, Wiley and Google Scholar) using the keywords ‘artificial intelligence’, ‘deep learning’, ‘machine learning’, ‘glioma’, ‘glioblastoma’, ‘radiomics’, ‘radiogenomics’ and ‘neurosurgery’. The Preferred Reporting Items for Systematic Reviews and Meta-Analyses (PRISMA) guidelines were followed, as illustrated in Fig. 1. The included articles were full-text articles in the English language published from inception until June, 2022. The excluded articles were review articles, abstracts only, letters to the editor, commentaries, as well as non-human, non-glioma, non-GB and non-English articles. The duplicate articles were deleted. The articles were then retrieved, screened and then categorized according to the use of AI in glioma. Each article was subsequently reviewed individually, summarized and the results are presented in Table I and in the Results section below. This type of study did not require approval from a research ethics committee.

## Results

The majority of the articles were from the USA (n=18) followed by China (n=11). The number of articles increased annually, reaching the maximum number in 2022. The majority of the articles were investigating glioma as opposed to GB (grade IV glioma). The majority of the articles were about both LGG and HGG (n=23), followed by HGG/glioblastoma (n=13). Additionally, three articles were about LGG only, and one article was on HGG only; two articles not specify the grade.

The article titled ‘Automatic assessment of glioma burden: A deep learning algorithm for fully automated volumetric and bidimensional measurement’ written by Chang *et al* (14) had the highest sample size among the other included studies, reaching a sample size of 897 samples. The most commonly used AI modality was deep convolutional neural networks (CNNs) alone or in combination with other methods.

The articles discussing the use of AI in glioma were classified into detection, grading, prediction of the IDH genotype, MGMT promoter methylation status (which is a mutation associated with an improved prognosis), 1p19q codeletion, survival prediction, treatment response, pseudo-progression and progression and the glioma functional network. The results of the systematic review and summary are presented in the following paragraphs.

### Detection

Since gliomas are frequently characterized by radiographic evaluation and magnetic resonance imaging (MRI), the precision of imaging-based tumor feature recognition has increased, since traditional machine learning algorithms use human-designed feature extraction to separate tumor characteristics (15). Rajagopal (16) used the feature optimization technique to distinguish glioma from non-glioma brain MRI images using an optimum collection of features. In order to identify the tumor areas in the brain MRI image and distinguish them from surrounding areas, the segmentation method was performed, and he achieved 96.5% specificity, 97.7% sensitivity, and 98.01% accuracy (16). Another study employed the SegNet classification system on the HGG BRATS 2017 dataset and achieved 96.1% sensitivity, 96.5% specificity, and 96.4% tumor pixel segmentation accuracy (17). One of the issues with glioma detection is the presence of noise, as well as low-sensitivity border pixels (18). Mathiyalagan and Devaraj (19) recognized and eliminated the noise content in the original brain MRI image using a ridgelet filter and fuzzy logic was then used to detect the edges in the noise-removed image, and contrast adaptive local histogram equalization was applied to the edge-detected brain image to improve the edge pixels. The suggested technique obtained 97.65% sensitivity, 97.8% sensitivity and 98.78% accuracy on the HGG brain MRI images in the BRATS 2017 dataset (19). Another systematic review found that the most frequent type of algorithm used in glioma detection was neural networks. Algorithm accuracy varied from 0.75 to 1.00 (median, 0.96; 10 articles) (20). The assessment of reporting quality using the Transparent Reporting of a multivariable prediction model for Individual Prognosis Or Diagnosis (TRIPOD) criteria resulted in a mean individual TRIPOD ratio of 0.50 (standard deviation, 0.14; range, 0.37 to 0.85) (20). A standard MRI can make it challenging to distinguish between brain metastases and glioma, as imaging results in certain clinical circumstances can frequently be similar (21). Jekel *et al* (21) conducted systematic review and meta-analysis of a subset of qualifying studies that used machine learning models for the non-invasive distinction of glioma from brain metastases. The average area under the receiver operating characteristic curve (AUC) from 17 studies was 0.916±0.052, while the average sensitivity (n=16) and specificity (n=15) were 0.868±0.123 and 0.843±0.235, respectively (21). In a different study, the ipsi- and contralesional hemispheres of patients were compared using network measurements to identify structural connectivity deficits caused by gliomas (22). The results were then linked to neurological testing. Both short- and long-range connection patterns were shown to be differentially impaired depending on the site of the lesion. In contrast to the contralesional hemisphere, the ipsilesional hemisphere exhibited a reduced global and local efficiency, according to the network analysis, which is indicative of the degradation of information flow across various network areas (22).

Even for radiologists, radiomics has proven to be useful in identifying differential diagnoses that were challenging to make. Furthermore, research has evaluated the added value of radiomics compared to the eyes of radiologists (23). Multiple research projects set up AI approaches to distinguish glioblastomas from single-brain metastases. A previous study which employed support vector machine (SVM) data classification and post-contrast 3D T1W gradient-echo sequence radiomics, revealed an accuracy of 85% and an AUC of 0.96(24). Another study using multiple feature selection and classification techniques, including SVM and Lasso, along with contrast-enhanced images, obtained a good accuracy and an AUC performance of 0.90. Moreover, the best classifiers outperformed expert neuroradiologists in terms of clinical performance (25). Primary CNS lymphoma (PCNSL) may exhibit heterogeneous contrast enhancement and internal necrosis, similar to glioblastoma behavior (23). A previous study found that radiomics were able to discriminate PCNSL from glioblastoma based on the apparent diffusion coefficient with optimal performance on both internal (AUC, 0.984) and external (AUC, 0.944) validation sets (26). Glioblastomas were distinguished from tumefactive multiple sclerosis by another study that used dynamic texture parameter analysis to extract features from the first pass of the contrast phase of dynamic susceptibility contrast-enhanced perfusion maps (27). AI can be used to differentiate between four molecular subtypes of glioblastoma: Neural, proneural, mesenchymal and classical by using the transcriptomic profiling tool (28). Research based on standard MRI sequences that used SVM obtained an accuracy of 71% in distinguishing the four subtypes (29).

### Glioma grading

Glial tumor grading is critical for patient care. It has a major impact on the extent of surgical resection, the need for adjuvant therapy and overall patient outcomes (30). Zhuge *et al* (31) used the deep convolutional neural networks method for glioma grading. The suggested approaches were tested using 5-fold cross-validation on The Cancer Imaging Archive (TCIA) LGG data and the Multimodal Brain Tumor Image Segmentation (BraTS) Benchmark 2018 training datasets and they achieved sensitivity of 0.935, a specificity of 0.972 and an accuracy of 0.963 for the 2D Mask R-CNN based method, and sensitivity of 0.947, specificity of 0.968 and an accuracy of 0.971 for the 3DConvNet method (31). Another study used radiomics for distinguishing between grades II, III and IV; that study retrieved radiomics characteristics using conventional, diffusion and arterial spin labeling perfusion MRI (32). That study utilized an SVM classifier and achieved a high AUC of 0.97 and an accuracy of 98% (32). Another study used SVM in the grading of gliomas in 120 individuals. These researchers used SVM with the synthetic minority over-sampling technique (i.e., over-sampling the abnormal class and under-sampling the normal class) and achieved 94-96% accuracy in diagnosing both HGGs and LGGs (33). In a different study, three distinct classification techniques, including random forest (RF), K-nearest neighbor and SVM were compared (34). After pre-processing, the tumor area was extracted from post-processed images using the fuzzy C-means segmentation approach. Texture, local binary pattern and fractal-based characteristics were obtained using MATLAB software. Subsequently, using the grasshopper optimization algorithm, they found that the RF performed better than the other classification techniques, with an accuracy of 99.09% (34). This finding is supported by another study that found that the RF-derived optimum feature set offered higher grading outcomes than previous methods utilizing the SVM classifier (35). Hsu *et al* (36) merged the whole slide imaging (WSI) and multiparametric magnetic resonance imaging (mpMRI) data guided by a confidence index. Experiments conducted on the validation dataset for the CPMRadPath 2020 competition revealed that combined judgments from both modalities performed better in glioma classification than either WSI or mpMRIs used alone (36).

### Prediction of the IDH genotype

An enzyme involved in the Krebs cycle and the energy metabolism of the cell is termed IDH. Alpha-ketoglutarate is typically accumulated from isocitrate in IDH wild-type gliomas; however, in cases of IDH mutation, isocitrate transforms into 2-hydroxyglutarate (30). The prognostic and predictive significance of IDH mutation renders it one of the most significant molecular markers, with the prognosis and treatment sensitivity being better for the IDH mutant subtype (37). The WHO classification of Tumors of the CNS was updated in 2016 to include molecular status for identifying diffuse gliomas, such as IDH gene mutation and chromosomal 1p/19q codeletion (9). A previous study used short echo time (TE) proton MR spectroscopy (1H-MRS) at 3T to classify IDH and TERTp mutation-based subsets of gliomas (38). They achieved an accuracy of 88.39%, a sensitivity of 76.92% and a specificity of 94.52% for a TERTp mutation in primary IDH wild-type gliomas, and an accuracy of 92.59%, a sensitivity of 83.33% and a specificity of 95.24% for a TERTp mutation in primary IDH wild-type gliomas (38). Another study used 3D-CNN in 94 cases of IDH mutation to associate multiparametric imaging characteristics with glioma IDH mutations, and 120 wild-type gliomas exhibited a higher effectiveness, attaining 98% sensitivity, 97% specificity and an AUC of 99% (39). Nalawade *et al* (40) assessed three CNN models (Inception-v4, ResNet-50 and DenseNet-161) using T2-weighted (T2w) MRI data from 120 individuals diagnosed with HGGs and 140 individuals diagnosed with LGGs. The highest-performing model with minimum preprocessing steps was determined to be DenseNet-161 with a 5-fold cross-validation. That study achieved a mean slice-wise accuracy, sensitivity and specificity of 90.5, 83.1, and 94.8%, and subject-wise accuracy, sensitivity and specificity of 83.8, 83.5, and 83.5%, respectively (40). Another study that employed the voxel-wise clustering method examined 69 patients with treatment-naive diffuse glioma using diffusion-weighted imaging, fluid-attenuated inversion recovery, pH-sensitive amine chemical exchange saturation transfers MRI, and contrast-enhanced T1-weighted imaging at 3 T (37). The 10-class clustering method performed best in predicting IDH mutation status, with a mean AUC, accuracy, sensitivity and specificity of 0.94, 0.91, 0.90, and 0.91%, respectively (37).

### MGMT promoter methylation status

When the MGMT promoter, an enzyme involved in DNA damage and dealkylation, undergoes hypermethylation, there is a positive association between this mutation and improved treatment outcomes with temozolomide (41). Previous studies have used radiomics to non-invasively determine the methylation status of the MGMT promoter. Levner *et al* (42) used artificial neural networks (ANN) in conjunction with two-dimensional discrete orthonormal Stockwell transform (2D-DOST) to perform a texture analysis of T2, fluid attenuated inversion recovery (FLAIR) and T1 post-contrast MR images to predict 59 patients with newly diagnosed glioblastoma regarding their MGMT promoter methylation status. An 87.7% accuracy rate was attained in that study, which was obtained by the 2D-DOST in combination with ANN (42). The study by Korfiatis *et al* (43) tested and assessed three different residual CNNs without any prior tumor segmentation pre-processing to predict the MGMT status on 155 brain MR scans. ResNet50 (50 layers), ResNet34 (34 layers) and ResNet18 (18 layers) achieved an accuracy of 94.90, 80.72, and 76.75%, respectively (43). In addition, Chang *et al* (44) used the MRIs of 259 glioma patients and a CNN model to predict the methylation status of the MGMT promoter. The model attained a level of accuracy of 83.0% (44). Another study combined IDHmut and MGMTmet to establish a significant prognostic molecular marker for gliomas (45). That study analyzed 162 individuals with gliomas, comprising 58 patients with IDHmut and MGMTmet co-occurrence and 104 patients with other statuses. The effectiveness of the models produced by the tree-based pipeline optimization tool was evaluated using the AUC. Using shape and textual features from the Laplacian-of-Gaussian-filtered Gd-3DT1, the gradient boosting classifier was trained and performed best in 4-fold cross-validation (average sensitivity, 81.1%; average specificity, 94%; average accuracy, 89.4%; average kappa score, 0.76; average AUC, 0.951) (45).

Precision medicine, in which the course of treatment is tailored to the individual genetic profile and epigenetic signature of each tumor, is within reach when phenotypic, genotypic and epigenetic features are combined in glioblastoma diagnostics (46). The MGMT promoter's methylation status has key therapeutic implications in predicting response to alkylating chemotherapy. As a result, studies have used radiomics to determine the methylation status of the MGMT promoter (46). An example is a study that used conventional MRI in order to anticipate the MGMT promoter methylation status in patients with glioblastoma. A subset of six features was used by the final random forest classifier and achieved an AUC of 0.88(47). Another study used a subset of 36 characteristics, in both the validation and test datasets, the final model achieved diagnostic accuracy of 87 and 80%, respectively (48). In addition, another study took advantage of positron emission tomography (PET) scans of 107 patients by the extraction of >1,500 features; it made use of a support vector machine classifier. In training, the model achieved an AUC of 0.94, and in testing, it reached 0.86(49).

### 1p19q codeletion

Oligodendroglioma is defined by IDH mutant gliomas containing 1p19q codeletion. Astrocytomas are the term for non-codeleted 1p19q tumors, whether or not they have an IDH1/2 mutation (50). Chang *et al* (44) achieved a 92% accuracy in predicting 1p19q codeletion status using a 2D/3D hybrid. Shofty *et al* (51) used 152 radiomics variables from conventional MRI to create an ensemble bagging tree classifier that identified a chromosomal 1p/19q co-deletion and obtained an accuracy of 87% following 5-fold cross-validation. Ge *et al* (52) augmented the data and employed contrast-enhanced T1 and T2-weighted MRs to obtain an accuracy of 89.39% on a cohort of 159 patients using a unique multistream deep CNN (a 7-layer 2D CNN). Han *et al* (53) developed a radiomics signature from 277 patients with WHO grade II and III gliomas using conventional MRI using a RF classifier. In the training cohort, the final model had an AUC of 0.89 while in the test cohort, it had an AUC of 0.76. They found that a combination model that included radiomics and clinical characteristics did not enhance the prediction of the chromosomal 1p/19q co-deletion (53). Akkus *et al* (54) used a multiscale CNN in 159 cases, they could predict the 1p19q codeletion status with an accuracy of 87.70%.

### Survival prediction

The prediction of the survival of patients with glioma has also been proposed using deep learning-based radiomics models. Chen *et al* (55) developed a machine-learning model for patients with HGG by obtaining eight clinical variables and 39 dose-volume histogram parameters. Their study used Cox proportional hazards, SVM and random survival forest (RSF) models. The RSF model outperformed the other two models, where the concordance indices of the training and testing sets were 0.824 and 0.847, respectively (55). The AUCs of the testing set for 1-, 2- and 3-year survival were 92.4, 87.7 and 84.0%, respectively. They demonstrated that with this approach, overall survival could be predicted and patients with HGG could be categorized into high- and low-risk groups (55). Another study combined an SVM technique with a deep learning architecture (56). A three-dimensional CNN was used in this deep learning architecture to extract distinguishing characteristics of brain tumors that already existed in conjunction with SVM; the two-step technique predicted the OS of 69 individuals with gliomas of high grade with an accuracy of 89% (56).

Recently, OS has been demonstrated to be predicted by radiomic models in patients with glioblastoma using radiomics features and clinical non-imaging data, such as age, resection status and the survival duration of patients (57). A previous study employed the bioinspired optimization approaches, genetic algorithms (GA) and particle swarm optimization (PSO) algorithms, to a fused feature set in the prediction of patient survival (OS) duration for the survival groups in the two and three classes (58). The technique obtained an AUC of 0.66 when using fusion feature + SVM + GA (3-class group) and 0.70 when using fused feature + SVM + PSO (2-class group) (58). Another study used radiomics nomograms along with radiomic signatures, ependymal and pia mater involvement (EPI), and age for the prediction of OS and dividing up patients with GBM into long-term vs. short-term survival (59). The nomogram, radiomic signature, age, and EPI accuracy for the external validation set were represented by the ROC curves as follows: 0.858, 0.826, 0.664 and 0.66(57). Another study used single feature class models, such as ‘clinical’, ‘pathological’, ‘MRI-based’ and ‘FET-PET/CT-based’ models and combinations, as well (60). The results of that study revealed that the MRI-based model improved performance over all single-feature class models and provided the optimal OS prediction performance when all features were combined. Adding treatment information improved prognostic performance even further, reaching C-indices of 0.73 (0.62-0.84) and 0.71 (0.60-0.81) on the validation set (60). Kickingereder *et al* (61) demonstrated the utility of radiomics in addition to the well-established prognostic indicators of age, surgical extent, Karnofsky performance score and the methylation status of the MGMT gene. When paired with clinical data, radiomic analysis enhanced progression-free survival and OS prediction using standard-of-care imaging (C-index increased from 0.637 to 0.696) (61).

### Treatment response

Neuro-oncology specialists frequently manually calculate tumor size using the two-dimensional diameters of the growing tumor. However, this method is only limited to interobserver variability and is time-consuming (14). Chang *et al* (14) created an automated algorithm that segments FLAIR hyperintensity and contrast-enhancing tumors, measuring tumor volumes and generating the largest bidimensional diameters possible in order to satisfy the Response Assessment in Neuro-Oncology (RANO) requirements (AutoRANO). The intraclass correlation coefficient for the FLAIR hyperintensity volume, contrast-enhancing tumors volume and RANO measures were used to compare longitudinal changes in tumor burden that were computed manually and automatically, respectively, 0.917, 0.966 and 0.850(14).

### Glioma functional network

In order to determine the functional network of gliomas, Xiang *et al* (62) attempted to create a systematic method for combining information with high throughput CRISPRCas9 tests for screening using machine learning techniques. They demonstrated that the network highly enhanced different biological pathways and may contribute to the development of gliomas. They also identified 12 putative Wnt/-catenin signaling pathway targeted genes, such as AARSD1, HOXB5, ITGA6, LRRC71, MED19, MED24, METTL11B, SMARCB1, SMARCE1, TAF6L, TENT5A and ZNF281, from densely coupled glioma functional modules (62). The overall survival prognosis of gliomas was highly associated with Cox regression modeling using these targets. Additionally, TRIB2 was identified as a glioma neoplastic cell marker by single-cell RNA-seq analysis of GBM samples (62).

### Tumor progression and pseudo-progression

True progression and pseudo-progression are difficult to reliably distinguish by MRI, and this continues to be a challenging issue in patient care despite the use of several methods, such as MR perfusion and watchful waiting. It is more challenging to identify pseudo-progression from true progression, as inflammatory responses with complex signal features have evolved with the emergence of novel immunotherapies (30). Since distinguishing glioblastoma tumor progression and pseudo-progression is difficult for the radiologist's view, a multivariate study demonstrated radiophenotypic signals differentiating between the two groups (63). In a leave-one-out cross-validation, the proposed signature predicted pseudo-progression with an accuracy of 87% (AUC, 0.92) and true progression with an accuracy of 84% (AUC, 0.83) (63).

## Discussion

The results of the reviewed studies demonstrate that, in comparison to other high-performing algorithms, deep learning methods can be dominant. Therefore, it is reasonable to believe that deep learning will survive and its product line will continue to grow. Future developments with deep learning indicate greater promise in various medical domains, particularly in the area of medical diagnosis. However, it is currently unclear whether deep learning can take the place of clinicians or doctors in the diagnosis of medical conditions (65). Studies on gliomas and glioblastomas have demonstrated a marked increase in the use of AI over the past few years for a variety of purposes, including diagnosis, grading, the prediction of molecular markers such as IDH genotype and MGMT promoter methylation status, prediction of survival, response to treatment, and even understanding functional networks. Each phase of the process of caring for patients with gliomas, including intraoperative tissue analysis, outpatient and oncology care, the post-operative acute phase, and intraoperative workflow analysis, could be completely transformed by artificial intelligence. AI can also improve brain tumor research and therapy and impact national guidelines and policies (65). AI models have successfully associated multiparametric imaging features with IDH mutations with high sensitivity and specificity, providing useful information for prognostic and treatment decisions. Moreover, the methylation status of the MGMT promoter must be known in order to assess the treatment efficacy. AI-based radiomics techniques have demonstrated efficacy in accurately estimating the methylation status, thereby facilitating customized treatment planning and the assessment of therapeutic outcomes. It should be mentioned that accurate generalizability estimation requires the validation of studies in independent, representative and clinically applicable datasets (21). In addition, the description of algorithm fine-tuning using data that is distinct from the training data should be saved for validation. Testing is the term for the objective assessment of an algorithm with data that is not included in the training or validation sets. Each study provided training and testing data; however, according to Subramanian *et al* (20), a number of them mislabeled testing as validation. Numerous studies are conducted based on small sample sizes or small databases. A related issue with small datasets is the well-documented ability of a multimillion-parameter deep learning algorithm to overfit to a single training cohort, leading to artificially inflated algorithm accuracy. With the comparatively small number of carefully selected datasets that are currently used in radiography research (15), a number of limitations may interfere with the clinical implantation of AI in the clinical setting. The adoption of the system depends on how well it fits into the radiologist's workflow, which includes the dictation software, picture archiving and communication system, and electronic medical records. Furthermore, manual intervention and the application of a range of tools are necessary for many segmentations and radiomic methods. To increase the generalizability of the performance of an algorithm across various imaging sites, acquisition parameters and patient populations, bigger and more diverse data sets may be required (15,66). Furthermore, there may be discrepancies in the current analysis of retrospective data obtained during standard clinical care. Ultimately, even with the ability to aggregate large cohorts from multiple hospitals, annotation remains a laborious process requiring a high degree of skill. Future developments of tailored semi-automated labeling tools and iterative re-annotation strategies may offer an efficient solution as manual annotations are frequently time-consuming (15). Finally, it has been noted that datasets from different global locations exhibit significant heterogeneity due to regional differences as there are different patient populations and pathologies in tertiary or academic medical centers, small hospitals and outpatient practices, and other diverse healthcare settings (57).

As regards limitations, even though applying AI methodologies to imaging analysis has produced positive results, there are still issues that need to be taken into account. These include the various imaging platforms, the various protocols and parameters used to obtain the images, the various patient classification criteria, and the variety of patient demographic and treatment data (64).

In conclusion, despite all the limitations and challenges associated with the use of AI and the validity and general globalization of its results, the use of AI in glioma is promising, and the variety in the application predicts a great future. It is applied in various steps of care ranging from diagnosis, grading, the prediction of prognosis, and reaching to treatment and post-operative care. There is a need for international collaboration in this field to share knowledge and experience to overcome the faced challenges. The present review explored the uses of AI in glioma, providing valuable insight for a broad audience in the neurological field. Neurologists, neurosurgeons, imaging specialists, pathologists and other healthcare professionals may find the discussion of current advances in AI integration in glioma care informative for their practice and future research endeavors.

## Figures and Tables

**Table I:** Articles discussing the applications of AI in glioma and glioblastoma.

Study no.	Author(s), year of publication	Country	AI uses	Sample size	Modality of AI	Validation methods	Tumor type	WHO grade	Accuracy, performance, precision	Future direction, Improvement methods	(Refs.)
1.	Akkus *et al*, 2017	USA	Predicting 1p/19q	159	Convolutional neural networks (CNN)	Data sets for testing, validation, and training separated out. As a test set, 90 slices were initially chosen at random from the data; these slices were never seen by the CNN while it was being trained. During training, 20% of the training set was divided into a validation set. Until training and validation accuracy became consistent, multiple configurations of a multi-scale CNN architecture were evaluated.	Glioma	LGG	93.3% (sensitivity), 82.22% (specificity), and 87.7% (accuracy).	The technique has the potential to be enhanced and substituted for surgical biopsy and pathological analysis in the prediction of 1p/19q codeletion status.	(54)
2.	Alqazzaz *et al*, 2019	UK	Segmentation	285	Convolutional neural network SegNe	The accuracy of the classification was assessed using data from a 5-fold cross validation.	Glioma	LGG + HGG	F-measure scores of 0.85, 0.81, and 0.7	The training phase of the suggested method takes a long time, but additional post-processing techniques might increase the accuracy of our approach, and SegNet models could be saved as trained models and improved with the use of more training datasets. Consequently, in order to improve the suggested system's performance over time, a longitudinal study utilizing various FCN and CNN architectures should be conducted.	(17)
3.	Yogananda *et al*, 2021	USA	MGMT promoter methylation status determination	247	T2WI-only network (MGMT-net)	Three groups were formed from the randomly shuffled dataset subjects. To ensure that every fold of the cross-validation was a new training phase based on a distinct combination of the three groups, the three groups alternated between training, in-training validation, and held-out testing groups.	Glioma	LGG + HGG	Accuracy 94.73%, sensitivity and specificity of 96.31 and 91.66%	Employing a bigger sample size from several imaging facilities.	(39)
4.	Chang *et al*, 2019	USA	Segments abnormal fluid attenuated inversion recovery (FLAIR) hyperintensity, contrast-enhancing tumor, quantitating tumor volumes, and Response Assessment in Neuro-Oncology (RANO)	897	3D U-Net architecture	By comparing automated measurements to manual measurements obtained from experts, the algorithm's performance was validated in two patient cohorts: a longitudinal postoperative patient cohort from a single institution, and a multi-institutional pre-operative patient cohort.	Glioma	LGG + HGG	Intraclass correlation coefficient (ICC) ranged from 0.850 to 0.991	Before it is widely used, multicenter clinical trials will be required for additional validation.	(14)
5.	Chang *et al*, 2018	USA	Predict molecular genetic mutation status	259	Convolutional neural network	Eighty percent of the data were used for training, and twenty percent were used for validation. Following fivefold cross-validation, only the patients in the validation cohort have their results published.	Glioma	LGG + HGG	Accuracy ranged from 83 to 94%	Subsequent research endeavours will require augmenting the training set to encompass an array of cancer sites and MR imaging scanners.	(44)
6.	Chato and Latifi, 2021	USA	Developing automatic overall survival time prediction system (OST)	335	Neural network and feature fusions	Three sets of the dataset are separated out: training, validation, and testing. Model parameters were adjusted using the validation data to look for overfitting or bias in the model.	Glioma/glioblastoma	LGG + HGG	Accuracy ranged from 63.2 to 65.1%	Expanding the dataset, utilizing a balanced dataset, utilizing the resection status feature's full information, merging shape and texture features with volumetric and location features, utilizing a multimodal dataset, and creating an accurate OST regression model.	(57)
7.	Chen *et al*, 2021	China	Survival prediction	95	Random survival forest (RSF), support vector machine (SVM), and Cox proportional hazards (CPHs) models	In the training set, a 5-fold cross-validation strategy was used, and the variable number was determined based on the cross-validation accuracy.	Glioma	HGG	Area under the curve ranged from 87.7 to 92.4%. concordance index ranged from 0.792 to 0.847	Since tumor heterogeneity and radiobiological factors were not found in the study, they should be taken into account in future EUD and TCP models. Future research should take into account radiomics features and molecular parameters found by conventional diagnostic techniques.	(55)
8.	Cho *et al*, 2018	Korea	Determine the glioma grading.	285	logistics, support vector machines, and random forest classifiers	The test and training data were separated using a five-fold cross validation.	Glioma	LGG + HGG	AUC ranged from 0.8866 to 0.9400	Utilizing data from a different clinical site for independent validation. strategies for oversampling minority classes.	(5)
9.	Fekonja *et al*, 2022	Finland	Characterize glioma related decreases in structural connectivity	37	Network-based statistics (NBS)	Outcomes and neurological evaluation were correlated.	Glioma	LGG + HGG	Results revealed hat lesion location resulted in differential impairment of both short and long connectivity patterns		(22)
10.	Ge *et al*, 2018	Sweden	Classification	N/M	Novel multistream deep Convolutional Neural Network (CNN)	During training, an early stopping strategy was used, wherein the network's parameters were fixed at a specific epoch once the best validation performance was attained.	Glioma	LGG + HGG	Accuracy ranged from to 89.39 to 90.87%	Larger glioma data sets require extensive testing.	(52)
11.	Hagiwara *et al*, 2022	USA	Differentiate isocitrate dehydrogenase (IDH) mutation status	69	Voxel-wise clustering method of multiparametric magnetic resonance imaging (MRI). The logarithmic ratio of the labels in each class within tumor regions was applied to a support vector machine	Leave-One-Out Cross-Validation (LOOCV)	Glioma	LGG	Mean area under the curve, accuracy, sensitivity, and specificity of 0.94, 0.91, 0.90, and 0.91, respectively.	To predict 1p/19q codeletion status using the same algorithm with a larger cohort, more research is necessary. substituting external validation for LOOCV.	(37)
12.	Hedyehzadeh *et al*, 2021	Iran	Grade detection	457	Fuzzy C-Means (FCM) segmentation method. Classification methods including Random Forest (RF), K-Nearest Neighbor (KNN) and Support Vector Machine (SVM)	4-Fold cross-validation technique	Glioma	LGG + HGG	Sensitivity ranged from 51.96 to 88.35, Specificity from 61.89 to 96.12, Accuracy from 59.96 to 99.09	Utilizing distinct cohorts for the test and training phases. The suggested approach's performance would be tested on various cohorts, which would increase the model's generalizability and produce more accurate results because of the image augmentation. using the TCIA's more recent glioma databases.	(34)
13.	Hsu *et al*, 2022	China	Glioma subtype classification	369	Hybrid fully convolutional neural network (CNN) and using the WSI-based approach and the mpMRIs-based approach	For the testing and validation sets in the CPM-RadPath 2020 challenge, there are 35 and 73 cases, respectively. The prediction results are submitted by participants to the challenge for an online algorithm evaluation.	Glioma	LGG + HGG	Balanced accuracy 0.654	Certain empirical parameters, like the number of extracting patches and the confidence index threshold, have an impact on the suggested method. To filter out the non-representative prototypes, the suggested method calls for the intervention of a qualified professional or expert.	(36)
14.	Jekel *et al*, 2022	USA	Differentiation of Glioma from Brain Metastasis	29 studies	Support vector machines (SVM), k-nearest neighbors' algorithms (kNN), deep neural networks (DNN), or convolutional neural networks (CNN), and traditional ML algorithms.	The sum of all studies' internally validated performance metrics, which produced dichotomized models for classifying brain metastases and gliomas.	Glioma and brain metastasis	HGG/glioblastoma	Average AUC 0.916±0.052, while average sensitivity and specificity were 0.868±0.123 and 0.843±0.235, respectively	The clinical application of machine learning for the distinction between glioma and brain metastasis depends on compliance with quality guidelines and validation on external datasets.	(21)
15.	Korfiatis *et al*, 2017	USA	Predicting methylation of the 6-methylguanine methyltransferase (MGMT) gene status	155	Residual deep neural network (ResNet)	It was used with stratified cross validation, a k-fold variant that yields stratified folds.	Glioblastoma multiforme	HGG/glioblastoma	Accuracy ranged from 76.75 to 94.90%	In order to fully assess the model's robustness, future work should incorporate datasets from several sites.	(43)
16.	Levner *et al*, 2009	Canada	Predicting the MGMT promoter methylation status	59	L1-regularized neural networks	Leave-one-out cross validation strategy (LOOCV)	Glioblastoma	HGG/glioblastoma	Accuracy of 87.7%		(42)
17.	Mathiyalagan and Devaraj, 2021	India	Detecting and classifying the glioma	342	Fuzzy C means algorithm and adaptive neurofuzzy inference system (ANFIS) classification method are used for segmentation.	Not mentioned.	Glioma	LGG + HGG.	Sensitivity ranged from 96.1 to 98.7, Specificity from 97.5 to 98.8, Accuracy from 98.1 to 99.8	As a future development of this manuscript, the deep learning model will be utilized to identify the tumor regions, negating the need for the pre-processing techniques described in this manuscript.	(19)
18.	Nalawade *et al*, 2019	USA	Predicting IDH status	260	Residual network (ResNet-50), densely connected network (DenseNet161), and Inception-v4.	Five-fold cross validation	Glioma	LGG + HGG.	Accuracy of 83.8%	This method can be extended in subsequent research to categorize IDH1 and IDH2 subtypes. multiparametric imaging data being incorporated into the training model.	(40)
19.	Nie *et al*, 2016	USA	Predict if the patient has a long or short overall survival (OS) time	69	Convolutional neural networks (CNNs), a multi-channel CNNs (mCNNs), Support Vector Machine (SVM)	10-fold cross-validation	Glioma	LGG + HGG	Accuracy ranged from 62.96 to 89.9%		(56)
20.	Ozturk-Isik *et al*, 2019	Turkey	Classify the IDH and TERTp mutational status	112	SVM, decision trees, and k-nearest neighbor (kNN) algorithms	Not mentioned.	Glioma	LGG + HGG	Sensitivity ranged from 76.92 to 83.33%, Specificity from 94.52 to 95.24%, Accuracy from 88.39 to 92.59%	Additional methods for improving the quantification of 2HG include the use of 2D correlation spectroscopy techniques, spectral editing, and a longer echo time of 97 msec with PRESS. Image guided biopsy can be used for validation. utilizing extra genetic markers.	(38)
21.	Rajagopal, 2019	India	Brain tumor detection and segmentation with optimized ant colony features	100	Random forest classifier	Not mentioned.	Glioma		97.7% of sensitivity, 96.5% of specificity, and 98.01% of accuracy		(16)
22.	Sengupta *et al*, 2019	India	Grading	53	Support vector machine (SVM) classifier	12-fold cross-validation (CV), Held-out validation	Glioma	LGG + HGG	Classification error for Grade II vs. III was 3.7%, for Grade III vs. IV was 5.26%, and for Grade II vs. III vs. IV was 9.43%	In these situations, signal intensity curves of notable vessels can be used as a distinguishing characteristic. bigger data set with grade I patients included.	(35)
23.	Shofty *et al*, 2017	Israel	Classification of patients with LGG and IDH mutation	47	Support vector machines (SVM), Nearest neighbor classifiers (kNN), Ensemble classifiers	Five-fold cross-validation	Glioma	LGG	Sensitivity=92%, specificity=83% and accuracy=87%, and with area under the curve = 0.87.	Further research involving a larger patient population is required to thoroughly examine the role that location characteristics play in the classification of cranial lesions.	(51)
24.	Subramanian *et al*, 2021	USA	Identification of Gliomas	12 articles (median sample size: 280 patients)	Neural network and Support vector machine	Using exclusive image sets from the same dataset for testing and training (7 articles) and five-fold cross validation (5 articles).	Glioma	LGG + HGG	Accuracy ranged from 0.75 to 1.00 (median 0.96), Dice coefficient ranged from (0.92 to 0.98)	Future research would benefit from focusing more on reporting standards quality and employing external datasetsfor algorithm testing.	(20)
25.	Tian *et al*, 2018	China	Grading	153	Support vector machine (SVM) classifiers	10-Fold cross validation	Glioma	LGG + HGG	AUCs were 96.8%/0.987 for classifying LGGs from HGGs, and 98.1%/0.992 for classifying grades III from IV	Involves molecular changes like 1p/19q codeletion and IDH mutation. employing an independent validation cohort.	(32)
26.	Xiang *et al*, 2022	China	Predicting and detecting glioma functional network	67 glioma cell lines, and 959 genes.	Random forest (RF), Multivariate Adaptive Regression Splines (MARS), Support Vector Machines with Radial Basis Function Kernel (svmRadial), Weighted kNearest Neighbor Classifier (kknn), and ClusterONE algorithm	Ten iterations of fivefold cross validation were conducted in order to fine-tune the parameters.	Glioma	LGG + HGG	The area under the ROC curves of 0.94	The single-cell RNA-seq data analysis revealed the presence of TRIB2, a glioma neoplastic cell marker that may be targeted by medication to address tumor heterogeneity issues.	(62)
27.	Zhang *et al*, 2020	China	Predict the co-occurrence of IDHmut and MGMTmet	162	Tree-based pipeline optimization tool (TPOT)	Inner 10-fold cross validation Ind an outer 4-fold cross validation	Glioma	LGG + HGG	(Average sensitivity=81.1%, average specificity= 94%, average accuracy=89.4%, average kappa score=0.76, average AUC=0.951).	It should be more powerful and a wiser decision to include more patients with varying scanners in the future. making use of a separate, external validation dataset. Features such as diffusion tensor imaging, dynamic susceptibility contrast perfusion-weighted imaging, and proton MR spectroscopy can be extracted from advanced magnetic resonance sequences. Also it would be worthwhile to investigate grouping cases into four categories in the future: IDHmut and MGMTmet, IDHwt and MGMTunmet, IDHwt and MGMTmet, and IDHmut and MGMTunmet.	(45)
28.	Zhang *et al*, 2017	China	Grading	120	25 WEKA classifiers	Leave-one-out cross validation (LOOCV)	Glioma	LGG + HGG	Accuracy ranged from 0.945 to 0.961	To prevent the imbalanced sample problem, a large number of balanced sample data can be incorporated into the model construction process. To enhance the model's capacity for generalization, a two-fold cross-validation approach and additional validation using samples gathered from separate institutions can be carried out. Finally, future research can incorporate the deep learning technique.	(33)
29.	Zhuge *et al*, 2020	USA	Grading	285	Deep convolutional neural networks (CNNs).	Five-fold cross validation	Glioma	LGG + HGG	Sensitivity ranged from 0.935 to 0.947, Specificity from 0.968 to 0.972, Accuracy from 0.963 to 0.971	Looking into the use of deep CNNs to combine digital pathology images with images from conventional, advanced MRI modalities to achieve more precise glioma grading.	(31)
30.	Akbari *et al*, 2020	USA	Discrimination between trueprogression and pseudo-progression	63	Convolutional neural network (CNN)	Leave-one-out-cross validation (LOOCV), and inter-institutional validation	Glioma	HGG/glioblastoma	Accuracy ranged from 75 to 87%. With AUC from 0.80 to 0.92	Validation of data from many institutions. Increase the sample size.	(63)
31.	Artzi *et al*, 2018	Israel	Differentiation between glioblastoma and brain metastasis	439	Support-vector machine (SVM), k-nearest neighbor, decision trees, and ensemble classifiers		Glioma	HGG/glioblastoma	Mean accuracy from 0.75 to 0.90; sensitivity from 0.57 to 0.11; specificity from 0.76 to 0.99; and AUC from 0.57 to 0.98	The classification results may be greatly enhanced by dividing this group into subgroups based on the primary tumor origin and greatly increasing the number of patients in each category. Future research ought to look at the classification outcomes in light of the total tumor volume.	(24)
32.	Das *et al*, 2021	India	Segmentation and overall survival period prediction	163	Random Forest, SVM, XgBoost, LGBM, and U-Net++	Five-fold cross validation	Glioma	HGG/glioblastoma	AUC from 0.63 to 0.70	Large datasets can be used to validate the method's robustness and performance, and advanced deep learning techniques can be applied to classification.	(58)
33.	Kang *et al*, 2018	Korea	Identifying atypical primary central nervous system lymphoma (PCNSL) mimicking glioblastoma.	112	Random forest classifier	Internal and external validation sets. 10-fold cross validation	GBM or PCNSL	HGG/glioblastoma	AUC from 0.787 to 0.983. Sensitivity (%) from 85.7 to 95.2. Specificity (%) from 96.7 to 97.8	Larger cohort research and imaging acquisition standardization.	(26)
34.	Kickingereder *et al*, 2016	Germany	Stratification and prediction of survival	119	Supervised principal component (SPC) analysis	10-fold cross validation	Glioma	LGG + HGG	(OS: IBS, 0.142; C index, 0.696; PFS: IBS, 0.132; C index, 0.637). OS=overall survival, IBS=integrated Brier scores	In the future, postprocessing time should significantly decrease with the use of customized high-performance and parallel computing. The validation of customized feature selection techniques is necessary for the unique kind of data that emerges from radiomic analyses. It is important to evaluate the connections between genomic sequencing and imaging signatures.	(61)
35.	Kong *et al*, 2019	China	Predicts MGMT promoter methylation status	107	Support vector machine (SVM)	Validation cohort	Glioma	LGG + HGG	(AUC) from 0.86 to 0.94. Accuracy from to 77.8 to 91.3%. Sensitivity from 75.0 to 94.9%. Specificity from 81.3 to 87.5%	Large patient cohort multicenter studies might be crucial to enhancing the prediction model's performance and generalizability. Additional research with extended follow-up times. Data from multimodality imaging (e.g. g. data from PET and MRI using different tracers) could be added to the radiomics model to predict the methylation status of the MGMT promoter in gliomas.	(49)
36.	Li *et al*, 2018	China	Prediction of MGMT methylation status	133	Boruta, and random forest	Independent validation cohorts	Glioma	HGG/glioblastoma	AUC=0.88, accuracy=80%	Combining dynamic contrast-enhanced (DCE) and DTI images. The model's potential clinical utility should be demonstrated using larger data sets from more institutes. Expanding the training data set can also significantly enhance the machine learning-based model's prediction accuracy.	(47)
37.	Peeken *et al*, 2018	Germany	Predict patients' overall survival (OS) and progression-free survival (PFS)	189	Multiple random survival forest prediction models	Internal validation	Glioma	HGG/glioblastoma	C-indices of 0.73 (0.62-0.84) and 0.71 (0.60-0.81) for OS and PFSS, respectively	Greater quantity of training sets and patients. Using dynamic FET-PET/CT measures or texture features could improve prognostic performance.	(60)
38.	Qian *et al*, 2019	China	Differentiating glioblastoma (GBM) from solitary brain metastases (MET)	412	Adaboost Classifier (Ada), k-nearest neighbor (KNN), multi-layer perceptron (MLP), decision tree (DT), naïve Bayes (NB), RF, and SVM.	5-fold cross-validation	Solitary brain MET and GBM	HGG/glioblastoma	(Area under the curve (AUC) ≥0.95 and relative standard deviation in percentile (RSD) ≤6)	The predictive effectiveness of radiomics will be further increased by using higher quality images and standardized protocols. By incorporating multi-model imaging data (diffusion tensor and perfusion imaging), the model can be enhanced.	(25)
39.	Rathore *et al*, 2019	USA	Classification of glioblastoma	112	Support ector machines	5-fold cross-validation	Glioma	HGG/glioblastoma	Accuracy from 75.9 to 88.4%, sensitivity from 71.4 to 83.9, specificity from 72.8 to 92.3		(29)
40.	Verma *et al*, 2017	Switzerland	Differentiating Enhancing Multiple Sclerosis Lesions, Glioblastoma, and Lymphoma	32	Dynamic texture parameter analysis (DTPA)				P-value between <0.00 and 0.05	Employing a bigger patient group.	(27)
41.	Xi *et al*, 2017	China	Predict MGMT methylation status	98	Support vector machine	Independent validation cohort. 10-fold cross-validation	Glioma	HGG/glioblastoma	Accuracy of 86.59%, sensitivity of 88.80%, specificity of 83.84%	More scanning sequences, such as fluid attenuated inversion recovery (FLAIR), DWI, and DCE with a larger patient sample will be included in future studies to improve the predictive accuracy and validate the radiomics features during treatment. In our upcoming work, we will also examine additional GBM molecules that have radiomics characteristics.	(48)
42.	Xu *et al*, 2021	China	Stratify GBM patients into long- vs. short-term survival.	158	LR, SVM, Bayes and K nearest neighbor algorithms, and random forests	External validation	Glioma	HGG/glioblastoma	Accuracy of 0.878 and 0.875, a specificity of 0.875 and 0.583, and a sensitivity of 0.704 and 0.833, respectively, in the training and test set	More subjects will need to participate in a prospective clinical study in order to fully comprehend the significance of EPIs in GBM patients' prognoses. utilizing one of the predictors, the Karnofsky Performance Scale.	(59)

AI, artificial intelligence; LGG, low-grade glioma; HGG, high-grade glioma; CNNs, convolutional neural networks; MRI, magnetic resonance imaging; MGMT promoter, O^6^-methylguanine-DNA-methyltransferase promoter; GBM, glioblastoma multiforme; DTPA, dynamic texture parameter analysis; RF, random forest; KNN, K-nearest neighbor; SVM, support vector machine.

## Data Availability

Data sharing is not applicable to this article, as no datasets were generated or analyzed during the current study.
